# Interactions between the Multicolored Asian Lady Beetle *Harmonia axyridis* and the Parasitoid *Dinocampus coccinellae*

**DOI:** 10.3390/insects7040067

**Published:** 2016-11-24

**Authors:** Maria Luisa Dindo, Santolo Francati, Alberto Lanzoni, Cinzia di Vitantonio, Elisa Marchetti, Giovanni Burgio, Stefano Maini

**Affiliations:** Department of Agricultural Sciences, Alma Mater Studiorum University of Bologna, Viale G. Fanin 42, 40127 Bologna, Italy; santolo.francati2@unibo.it (S.F.); alberto.lanzoni2@unibo.it (A.L.); cinziadv84@libero.it (C.d.V.); elisa.marchetti3@unibo.it (E.M.); giovanni.burgio@unibo.it (G.B.); stefano.maini@unibo.it (S.M.)

**Keywords:** *Harmonia axyridis*, *Dinocampus coccinellae*, *Adalia bipunctata*, exotic insect species, native parasitoids, host-parasitoid interactions, biocontrol

## Abstract

*Harmonia axyridis* (Pallas) has been introduced either intentionally or accidentally in different areas outside its native range, where it is often regarded as invasive. *Dinocampus coccinellae* (Schrank) has been recorded to parasitize *H. axyridis* in the field, both in the native and introduced areas, Italy included. The percent of parasitism found in our field investigation was low (four percent). The effect of exposure time of *H. axyridis* to *D. coccinellae* and the impact of parasitization on host longevity, oviposition capacity and egg fertility were evaluated in the laboratory. The acceptance and suitability of *H. axyridis* as host for *D. coccinellae* were then studied, in comparison with the native coccinellid *Adalia bipunctata* (L.), which shares the same ecological niche. The effects of parasitization on female longevity and reproduction capacity in the exotic vs. the indigenous lady beetle were also investigated. The overall results showed that *D. coccinellae* negatively affected the fitness of *H. axyridis*, more than that of *A. bipunctata*. The parasitoid may thus play a marginal role in controlling the populations of the Asian lady beetle, without representing a threat to *A. bipunctata*.

## 1. Introduction

The multicolored Asian lady beetle *Harmonia axyridis* (Pallas) (Coleoptera: Coccinellidae), an active predator of aphid and coccid pests originating from Asia [1], was introduced, either intentionally (as a biocontrol agent) or accidentally, in different areas outside its native range, including North America, South America, Europe and a few regions of Africa [2]. In the introduced environments, this lady beetle has spread at a very fast rate, probably due to its high survival, reproduction and dispersal capacity, and wide polyphagy [3,4,5]. In Italy, it was first recorded in 2006 [6]. From its establishment in different countries of introduction, adverse effects on native lady beetles have been shown, so the species is often regarded as invasive [7,8,9].

Recorded parasitoids of *H. axyridis* in native areas include the phorid fly *Phalacrotophora philaxyridis* (Disney) [10], tachinid fly *Medina luctuosa* (Meigen) and braconid wasp *Dinocampus coccinellae* (Schrank) [11]. Moreover, in the new areas some native parasitoids, such as the tachind *Strongygaster triangulifera* (Loew) in North America [12,13] and different phorid species in Europe [14,15], have been reported to attack *H. axyridis*. *Dinocampus coccinellae*, a cosmopolitan parasitoid of adult coccinellids, is the only hymenopterous species reported to date to parasitize *H. axyridis* both in native and introduced environments. Females usually reproduce by thelytokous parthenogenesis [16] and oviposit mostly in adult lady beetles, but, especially when adult hosts are scarce, larvae and pupae can also be parasitized [17,18]. Oviposition has to be very rapid to overcome host mobility and defensive reactions and the rate of parasitism may fluctuate considerably, depending on location, season and host species [16]. As a consequence of the parasitoid larval activity, the host gonads and fat body undergo great damage [19]. The lady beetles may, however, survive and even retain their ability to mate and lay eggs after parasitoid egression [20,21], but they are considerably weakened and almost paralyzed with parasitoid cocoon between their legs. Knowledge of the interactions occurring between *H. axyridis* and this or other native parasitoids in the areas of introduction deserves to be deepened, also considering that the impact of native natural enemies on introduced exotic insects may become more effective over time [22].

The first aim of this study was to find if any field-collected *H. axyridis* compared with native lady beetles was successfully parasitized by indigenous parasitoids of coccinellids in the Bologna area (Emilia Romagna region, northern Italy). Since only *D. coccinellae* was detected (see below), a number of interactions between this wasp and *H. axyridis* were studied in the laboratory, including the effects of host exposure time on parasitoid development. The effects of exposure to *D. coccinellae* on *H. axyridis* longevity, oviposition and egg fertility were also investigated. In the laboratory, Berkvens et al. [20] formerly showed a reduced reproductive capacity of *H. axyridis* that had all been stung by *D. coccinellae*. In our study, instead, either males or females were exposed to parasitoid and then paired with non-exposed adults of the opposite sex, in order to evaluate the effects of parasitization on male and female reproductive capacity separately. Finally the acceptance and suitability of *H. axyridis* vs. the native lady beetle *Adalia bipunctata* (L.) (Coleoptera: Coccinellidae) for *D. coccinellae* were investigated, as well as the effects of parasitization on female longevity, oviposition and egg fertility and data were used to compare the exotic vs. the indigenous coccinellid species. *Adalia bipunctata* was selected, because it shares the same ecological niche as *H. axyridis* and it is abundant in northern Italian agroecosystems [23].

## 2. Materials and Methods

### 2.1. Field Survey

Samples of *H. axyridis* and other coccinellid species, namely *Coccinella septempunctata* L., *A. bipunctata* and *Hippodamia variegata* Goeze, were field collected in the area surrounding the Department of Agricultural Sciences of the University of Bologna (DipSA) (Emilia-Romagna region, Italy, 44°48′39″ N, 11°37′84″ E). The samples were collected weekly from March to November 2010 on different trees (peach and apple trees, oaks and plane trees) but also bushes (roses and bay laurel) and grasses (sunflowers, wheat, nettle, etc.), and on building walls. All the specimens were collected, transferred to the laboratory and identified using the morphological characteristics described in Majerus and Kearns [24] and Koch [1]. They were then placed in plastic boxes (25 cm × 20 cm × 10 cm, 1 coccinellid species per box, a maximum of 30 individuals per box), with transparent lids (having holes covered by a steel mesh net) in order to check the possible emergence of parasitoids. The lady beetles were fed with frozen eggs of *Ephestia kuehniella* Zeller (Lepidoptera: Pyralidae) (purchased from Bioplanet, Cesena, Italy) [25], which were preferred to aphids (the standard food), because the eggs facilitated the parasitoid visual detection.

After a parasitoid (*D. coccinellae*, see Section 3.1 of the Results) was collected, it was isolated in a plexiglas cylinder (10 cm height, 9 cm diameter), placed at 26 ± 1 °C, 65 ± 5 r.h. and 16:8 L:D, and fed with tiny honey drops. For each host species, the results were evaluated in terms of number and percentages of successfully parasitized lady beetles (i.e., lady beetles that produced a parasitoid adult). The parasitoid species was identified by developmental, behavioral [16] and morphological characteristics [26]. Besides, cytochrome c oxidase subunit I (COI) sequences were checked against GenBank for highest similar matches by Umberto Bernardo and Liberata Gualtieri (Institute for Sustainable Plant Protection, National Research Council of Italy). Sequences showed an identity of 100% with *D. coccinellae* sequence. Voucher specimens were deposited at the University of Bologna, DipSA, Entomology area.

### 2.2. Insect Rearing

The insect rearing and laboratory tests were all performed at 26 ± 1 °C, 65 ± 5 r.h. and 16:8 L:D.

A colony of *H. axyridis* (*succinea* form) was established in April 2010 starting from eggs laid by females collected from the field, in the area surrounding DipSA, as in Section 2.1. A colony of *A. bipunctata* was established in 2010 from individuals collected in an organic peach orchard placed in Budrio (Bologna) (44°32′16″ N 11°32′04″ E). Field-collected specimens of the two coccinellid species were regularly added to the laboratory colonies to maintain genetic diversity. Both colonies were maintained in the laboratory of DipSA using the same procedure. Namely, both larvae and adults were fed ad libitum with aphids (*Myzus persicae* (Sulzer)), which were in turn reared on pea plants (*Pisum sativum* L.). The larvae were kept in plastic boxes (25 cm × 20 cm × 10 cm), whereas the adults were maintained in plexiglas cages (40 cm × 30 cm × 30 cm). In September 2010, after *D. coccinellae* started emerging from field-collected *H. axyridis*, a laboratory colony of the parasitoid was also established, using the Asian lady beetle as host. The parasitoid adults were maintained in a plexiglas cage (40 cm× 30 cm× 30 cm) and fed with tiny honey drops. At the beginning, *H. axyridis* adults were randomly exposed to *D. coccinellae* and kept in the cage until the detection of parasitoid cocoons. Emerged parasitoids were maintained in the same cage. The parasitoid rearing technique was refined following the results achieved in the experiment described below (see Section 3.2 of the Results). Namely, adult *H. axyridis* were exposed to *D. coccinellae* females for 1 h (10 coccinellids/female) and then moved to the plastic boxes until parasitoid cocoon formation. The lady beetles were fed with *E. kuehniella* eggs ad libitum. The newly formed cocoons were collected and transferred to plexiglas cages for emergence.

### 2.3. Effect of Exposure Time on the Development and Yields of D. Coccinellae in H. Axyridis

The experiment was performed to study the influence of exposure time of *H. axyridis* to *D. coccinellae* on the rates of parasitism and parasitoid development time. Newly formed *D. coccinellae* cocoons were collected from the stock colony and placed singly in plexiglas cylinders (10 cm height, 9 cm diameter). The parasitoid females were used for the experiment 2–4 days after emergence [20]. Three exposure time treatments of *H. axyridis* adults (4–6 day old) to *D. coccinellae* females were tested, namely 5 min, 1 h, 24 h. The 5-min and 24-h times were respectively selected following the experiment performed by Berkvens et al. [20] and the rearing procedure adopted by Firlej et al. [18], whereas the 1-h time was chosen by us for comparison. For each treatment time, 10 lady beetles of mixed sexes were exposed to 1 parasitoid in a plexiglas cylinder (20 cm height, 9 cm diameter). After the exposure, the lady beetles were removed from the cylinder, placed in a plastic box (11 cm × 30 cm× 19 cm) and fed with frozen *E. kuehniella* eggs until cocoon formation or lady beetle death. The newly-formed cocoons were placed individually in plexiglas cylinders and checked daily until adult emergence. For every treatment time, a total of 100 *H. axyridis* were tested.

The following parameters were evaluated: (1) number and percentage (calculated over the number of lady beetles exposed = 100) of lady beetles that produced a parasitoid cocoon; (2) number and percentage (calculated over the number of cocoons) of emerged parasitoid adults; (3) development times (in days) from exposure to cocoon formation (a); from cocoon formation to adult emergence (b); and from exposure to adult emergence (c).

### 2.4. Effects of Exposure to D. Coccinellae on H. Axyridis Longevity, Oviposition and Egg Fertility

The experiments were aimed at investigating the effects of *D. coccinellae* on *H. axyridis* couples, when either males or females were exposed to the parasitoid.

#### 2.4.1. Male Exposure

About 80 *H. axyridis* pupae were collected from the stock colony and placed individually in plexiglas cylinders (8 cm height, 6 cm diameter). The newly emerged adults were sexed by the morphological characteristics described in McCormack et al. [27]. The trial was conducted using 20 *H. axyridis* males (4–6 day old), which were removed from the cylinders and individually exposed to single 2–4 day old *D. coccinellae* females in other plastic cylinders (10 cm height, 9 cm diameter). The exposure time was reduced by half (30 min) compared to the best time found in the previous experiment, because of the 1:1 ratio between hosts and parasitoids. After exposure, each male was transferred to another cylinder and paired with a same-age female that had not been exposed to *D. coccinellae.* The cylinder was lined with bubble wrap as an oviposition substrate [28]. Twenty couples formed by non-exposed males paired with non-exposed females were maintained as controls under the same conditions. All couples were fed ad libitum with *M. persicae* and monitored daily until 26 days after male exposure and couple formation (i.e., 7–9 days more than the average time from host exposure to *D. coccinellae* cocoon detection found in the previous experiment). For every treatment, each couple (1 per cylinder) was considered as a replicate.

For the result evaluation, the exposed males were divided into “successfully” and “unsuccessfully” exposed on the basis of whether or not they produced *D. coccinellae* cocoon. The following parameters were considered: (1) number and percentage of successfully exposed *H. axyridis* males; (2) male longevity from emergence in days; (3) pre-oviposition; and (4) oviposition duration in days (both durations were counted from couple formation); (5) number of eggs/female laid during the 10 days following the first oviposition (E_10_), to represent fecundity [29,30]; (6) egg fertility (% hatched eggs = hatched eggs/E_10_ × 100).

#### 2.4.2. Female Exposure

This experiment was carried out following the same method as that described in Section 2.4.1, except female lady beetles were exposed to *D. coccinellae* and paired with same-age males that had not been exposed to the parasitoid.

### 2.5. Effects of D. Coccinellae on H. Axyridis vs. A. Bipunctata

#### 2.5.1. Acceptance and Suitability

To test the acceptance of *H. axyridis* vs. *A. bipunctata*, for each species 10 adult lady beetles of mixed sexes were exposed together to 1 parasitoid in a Petri dish (3 cm height, 20 cm diameter) for 1 h. The lady beetles were fed ad libitum with *M. persicae* for ten days, a sufficient time for the parasitoid eggs to hatch into larvae at 25 °C [31,32]. Subsequently, the lady beetles were frozen at −18 °C for 24 h and finally dissected to detect parasitoid larvae.

To test suitability, 10 *H. axyridis* or 10 *A. bipunctata* were exposed to 1 *D. coccinellae* for 1 h, as in the acceptance experiment, but the lady beetles were maintained on *E. kuehniella* frozen eggs and monitored until adult parasitoid emergence.

In both tests 6 replicates, each comprising 10 lady beetles per species, were carried out.

Acceptance (a); and suitability (b) were respectively evaluated in terms of percentages of (a) lady beetles containing a parasitoid larva; and (b) lady beetles producing a parasitoid adult.

#### 2.5.2. Effects on Longevity, Oviposition and Egg Fertility

This experiment was carried out following the methods described in Section 2.4.1. Females of *H. axyridis* or *A. bipunctata* (20 per species) were individually exposed to *D. coccinellae* and paired with same-species males that had not been exposed to the parasitoid. Twenty couples of *H. axyridis* and 20 couples of *A. bipunctata* (formed by non-exposed females paired with non-exposed males) were maintained as control. For every treatment, each couple was considered as a replicate. The exposed females were divided into “successfully” and “unsuccessfully” exposed. The results were evaluated in terms of (1) number and percentage of successfully exposed females of the two species; (2) female longevity in days; the same parameters number (3–6) described previously (Section 2.4.1 and Section 2.4.2). These parameters were considered only for the couples including females that oviposited at least one egg.

### 2.6. Data Analysis

The STATISTICA software for Windows was used for all statistical analyses in the study [33]. In the field survey, the data were analyzed using a 4 by 2 contingency table to test the independence of coccinellid species and number of parasitoids that emerged. In the experiment studying the effect of exposure time on the development and yields of *D. coccinellae*, the number of lady beetles that produced a parasitoid cocoon and of emerged parasitoid adults were analyzed by means of 2 by 2 contingency tables. The one-way ANOVA or Kruskal-Wallis non-parametric test (when heteroscedasticity occurred) were used to analyze the development times. In the experiments studying the effects of exposure to *D. coccinellae* on *H. axyridis* longevity, oviposition and egg fertility, the data were analyzed by one-way ANOVA followed by Tukey’s HSD multiple range test or by Kruskal-Wallis test followed by non-parametric multiple comparisons when heteroscedasticity occurred. The data concerning the acceptance and suitability of *H. axyridis* vs. *A. bipunctata* were analyzed by Wilcoxon test. Finally, in the experiment studying the effects of exposure to *D. coccinellae* on longevity, oviposition and egg fertility of *H. axyridis* vs. *A. bipunctata*, the data for female longevity, pre-oviposition and oviposition duration and egg fertility were analyzed by a factorial ANOVA using coccinellid species and exposure effect as main factors [34]. The data concerning oviposition duration were subjected to rank transformation prior to analysis to make them homoscedastic. The number of eggs laid in 10 days (E_10_) were analyzed separately by species or exposure effect, by one-way ANOVA or Kruskal-Wallis test when heteroscedasticity occurred. No statistical analysis was performed for the percentages of successfully exposed females, because no parasitoid cocoons were obtained from *A. bipunctata*. In all experiments the percentage values were arcsine transformed before analysis.

## 3. Results

### 3.1. Field Survey

**Figure 1:**
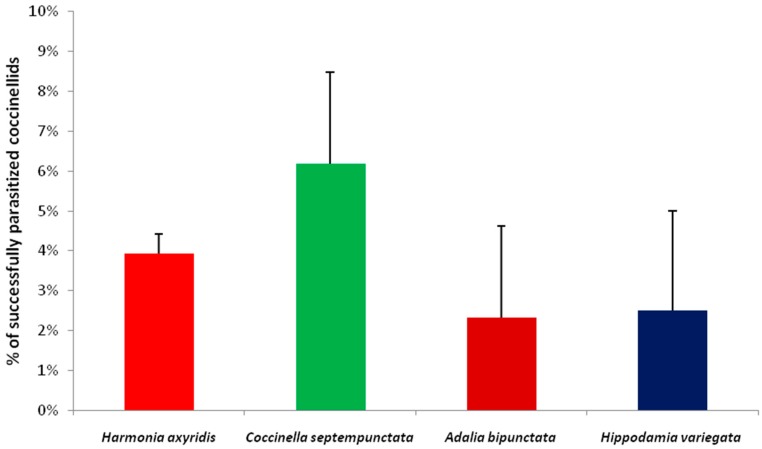
Effect of coccinellid species on parasitism by *D. coccinellae*. The coccinellids, *H. axyridis* (*n* = 1348), *C. septempunctata* (*n* = 113), *A. bipunctata* (*n* = 43) and *H. variegata* (*n* = 40) were field-collected from March to November in the year 2010 in the Bologna area (Emilia Romagna region, northern Italy). Columns indicate the percentage (±SE) of successfully parasitized coccinellids (i.e., coccinellid specimens from which a *D. coccinellae* cocoon emerged). See text for statistics.

*Dinocampus coccinellae* was the only parasitoid species obtained from the field-collected specimens. The rates of parasitism were very low, ranging from 2.3% to 6.2%. *Harmonia axyridis* was the most abundant coccinellid species. An overall number of 1348 individuals (1169 adults and 179 larvae/pupae) was collected. A total number of 53 *D. coccinellae* emerged from adults, and in only one case did a parasitoid emerge from an adult collected as a larva. *Dinocampus coccinellae* emerged also from the other field-collected adult coccinellid species, namely *C. septempunctata*, *A. bipunctata* and *H. variegata*. *Coccinella septempunctata* (Figure 1). No significant difference in rates of parasitism was found among the four coccinellid species (χ^2^ = 1.97, df = 3, *p* = 0.584).

### 3.2. Effect of Exposure Time on the Development and Yields of D. Coccinellae in H. Axyridis

The highest percentage of lady beetles that produced a parasitoid cocoon (36%) occurred when *H. axyridis* was exposed to *D. coccinellae* for 1 h compared to 5 min or 24 h, but the parasitoid adult emergence from cocoons was independent from exposure time (Table 1). The time from cocoon to adult emergence was also not significantly affected by exposure time. The time from exposure to parasitoid cocoon detection was, however, significantly longer for the parasitoids obtained following an exposure time of 24 h, which ultimately resulted in a significantly longer total development time for these individuals (Table 2).

### 3.3. Effects of Exposure to D. coccinellae on H. axyridis Longevity, Oviposition and Egg Fertility

#### 3.3.1. Male Exposure

Of the exposed males, 35% were exposed successfully (i.e., they produced a parasitoid cocoon). Exposure to *D. coccinellae* significantly influenced male longevity (H = 10.31, *n* = 40, *p* = 0.006). For the unsuccessfully exposed males, longevity (21.92 ± 0.69 days, *n* = 13) was significantly shorter than that of the control males (24.85 ± 0.56 days, *n* = 20). Both values were, however, not significantly different from the longevity of the succesfully exposed males (23.29 ± 0.94 days, *n* = 7). When the data for the successfully and unsuccessfully exposed males were pooled, the longevity (22.4 ± 0.78 days) was significantly shorter than that of the control males (H = 10.02, *n* = 40, *p* = 0.002). Therefore, exposure, whether successful or not, negatively influenced male longevity.

When *H. axyridis* females were paired with males either successfully or unsuccessfully exposed to *D. coccinellae*, the pre-oviposition and oviposition duration, and the number of eggs laid in 10 days (E_10_) were not significantly affected. All females were able to lay at least one fertile egg, but fertility (% hatched eggs) was significantly lower for the females paired with unsuccessfully exposed males compared to the control couples (Table 3).

#### 3.3.2. Female Exposure

Only 15% of the exposed females produced a parasitoid cocoon. Mean survival times (±SE) were 25.3 ± 0.62, 23.76 ± 0.67 and 21.67 ± 1.59 days for the non-exposed (*n* = 20), unsuccessfully exposed (*n* = 17) and successfully exposed (*n* = 3) females, respectively. The difference among treatment times was not significant (H = 2.11; *n* = 40; *p* = 0.35).

All females (either exposed or not) were able to oviposit. All eggs laid by the non-exposed females were fertile. Conversely, two (out of 17) unsuccessfully exposed females and two (out of three) successfully exposed ones were not able to produce fertile eggs. Exposure had no significant effect on pre-ovipostition duration, but the other parameters (oviposition duration, E_10_, egg fertility) were significantly lower for the successfully and unsuccessfully exposed females, compared to the controls (Table 4).

### 3.4. Effects of D. Coccinellae on H. Axyridis vs. A. Bipunctata

#### 3.4.1. Acceptance and Suitability

**Figure 2:**
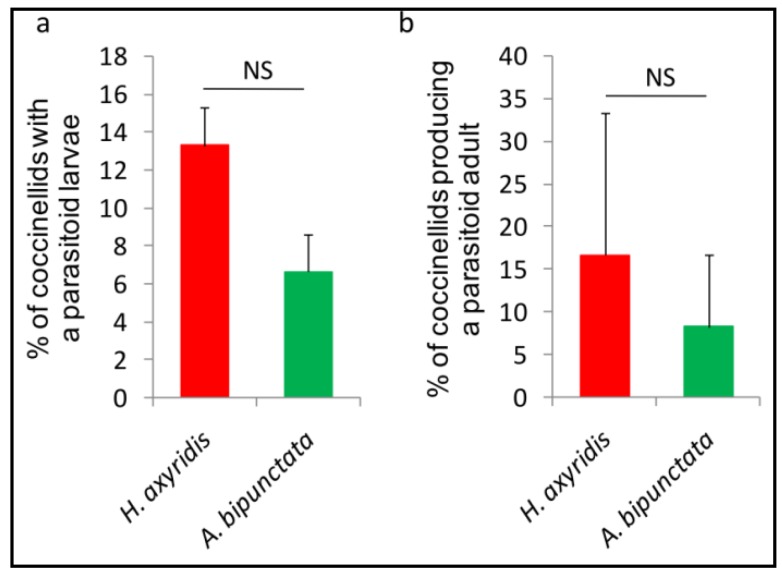
Acceptance (**a**); and suitability (**b**) of *H axyridis* vs. *A. bipunctata* by *D. coccinellae* (*n* = 6 for both tests). Percentage (mean ± SE) of lady beetles containing a parasitoid larva (**a**); or producing a parasitoid adult (**b**). See text for statistics.

The percentage of lady beetles containing a parasitoid larva (reflecting host acceptance) was not significantly different between the exotic and the native coccinellid species (*z* = 1.21, *p* = 0.225; *n* = 6) (Figure 2a). Differences for the percentages of lady beetles producing a *D. coccinellae* adult was also not significant between *H. axyridis* and *A. bipunctata* (*z* = 0.447, *p* = 0.655; *n* = 6) (Figure 2b). This parameter reflected the suitability of the two coccinellid species for the complete development of *D. coccinellae*.

#### 3.4.2. Effects on Female Longevity, Oviposition and Egg Fertility

Of the exposed *H. axyridis* females, four (20%) produced a *D. coccinellae* cocoon. All of these females oviposited (mean E_10_ ± SE = 61 ± 19.8), but only two were able to produce fertile eggs. One non-exposed (control) female per species and two and one unsuccessfully exposed *H. axyridis* and *A. bipunctata* females, respectively, failed to oviposit.

No *A. bipunctata* was exposed successfully to the parasitoid in this experiment. Therefore, for both coccinellid species, only the control and unsuccessfully exposed females were considered for comparison. No significant interaction of coccinellid species and exposure to *D. coccinellae* was found for any of the parameters analysed. Neither species nor exposure significantly affected longevity (Table 5), but pre-oviposition duration was shorter for the native compared to the exotic lady beetle. The effect of species, but not that of exposure, was significant for this parameter. The oviposition duration was not significantly influenced by coccinellid species, but it was significantly shorter for the exposed than for the non-exposed lady beetles (Table 5).

The total eggs laid in 10 days (E_10_) were 195.57 ± 49.2 and 325.11 ± 25.51 for the unsuccessfully exposed (*n* = 14) and the non-exposed (*n* = 19) *H. axyridis* females, respectively, and 151.32 ± 24.19 and 182.63 ± 19.5 for the unsuccessfully exposed (*n* = 19) and the non-exposed (*n* = 19) *A. bipunctata* females, respectively. The eggs laid by the unsuccessfully exposed females were significantly fewer than those laid by the non-exposed ones for the exotic, but not for the native coccinellid species (respectively F = 6.32; df = 1,31; *p* = 0.02 and F = 1.02; df = 36; *p* = 0.32). The difference between the eggs laid by *H. axyridis* and *A. bipunctata* was significant for the non-exposed (F = 19.68; df = 1,31; *p* = 0.00008), but not for the exposed lady beetles (H = 0.75; *n* = 33; *p* = 0.78). Egg fertilty was significantly affected both by coccinellid species and exposure (Table 5).

## 4. Discussion

*Harmonia axyridis* is the best known example of an exotic beneficial insect for which concerns have been raised over its invasiveness potential in introduction areas [2]. Sustainable management strategies are not developed for predatory coccinellids or other beneficial insects [35].

In our field survey, *H. axyridis* was the most abundant coccinellid species. It was attacked by *D. coccinellae*, which was already known as a parasitoid of the multicolored Asian lady beetle in other introductions, including United States [36], Canada [37], UK [38] and Belgium [20]. The Asian lady beetle was successfully parasitized at a low (4%) rate, although no significant differences in the parasitization occurred among the coccinellid species. A lower susceptibility of *H. axyridis* vs. *C. septempuntata* to *D. coccinellae* was previously shown [39], as well as the poor suitability of the other two species for this parasitoid [18,40]. Our field data suggest that, in accordance with previous studies [20,41,42], *H. axyridis* is a marginal host for *D. coccinellae* and, as a consequence, the parasitoid impact on the exotic lady beetle population is currently minimal. A comparison between the rates of parasitism by *D. coccinellae* of *H. axyridis* in diverse areas (either native or of introduction) is, however, difficult, because these rates are usually calculated in different ways by different authors [16].

The investigations carried out by de Castro-Guedes and de Almeida [43] showed that *H. axyridis* was also a poor host for *D. coccinellae* in the laboratory. The overall results of our study, however, indicated a better possibility of adaptation by *D. coccinellae* to *H. axyridis* in the laboratory than in the field. In the experiment aimed at evaluating the effect of exposure time on the development and yields of *D. coccinellae* in the exotic lady beetle, the rates of parasitism (which ranged from 18% to 36%) were higher than those found in wild samples. The greatest parasitoid yield was obtained with the exposure time of 1 h, which could represent the optimal time to maximize parasitoid production, at least with the host/parasitoid ratio used in this experiment (10:1). The 24-h and, especially, the 5-min treatments gave lower yields. The parasitoid development times were similar between the hosts exposed for 1 h and 5 min and were consistent with those observed, at 24 ± 2 °C, in *Coleomegilla maculata lengi* Timberlake, a suitable host for *D. coccinellae* [41]. Conversely, in the hosts exposed for 24 h, the time from exposure to cocoon detection and, consequently, the time from exposure to adult emergence were significantly longer. This result may be related to the parasitoid’s inability to discriminate between unparasitized and parasitized hosts [44]. Although *D. coccinellae* is a solitary parasitoid, superparasitism may occur and a single host may thus contain more than one egg and/or first instar larva, but only one larva survives to the second instar [45,46]. The delay in parasitoid development observed in the hosts exposed for 24 h (the longest exposure time tested) could, therefore, result from superparasitism and competition between first instar larvae [16,47,48].

In the experiment aimed at testing the effects of exposure to *D. coccinellae* on *H. axyridis* longevity, oviposition and egg fertility, both the male and female coccinellids exposed to parasitoid lived shorter than the controls, although, for females, no significant difference was found. This finding is in line with the observations reported by Hodek and Honěk [49] for other coccinellid species. Also, the reproductive capacity of the Asian lady beetle was affected by the exposure to *D. coccinellae*, as previously shown by Berkvens et al. [20]. In our study, however, the effects of exposure on males and females were investigated separately. In the females, apart from the pre-oviposition duration, all the considered parameters (e.g., oviposition duration, number of eggs laid in 10 days and egg fertility) were dramatically reduced in the successfully or even unsuccessfully exposed females (i.e., females exposed to *D. coccinellae* but not successfully parasitized). These data suggest that the parasitoid larval development, though incomplete (possibly due to an inhibition of the teratocyte development [41]), was sufficient to inhibit *H. axyridis* reproduction capacity [39].

Egg fertility was the only parameter negatively influenced by male exposure. The oviposition of unfertile eggs by the non-exposed females paired with exposed males was expected, since host castration was found to be induced by *D. coccinellae* [19] (though, according to Kadono-Okuda et al. [32], in the early phase of development, the parasitoid larvae may have a stimulatory effect on the maturation of the host’s female gonads).

The results of the tests aimed at evaluating the effects of *D. coccinellae* on *H. axyridis* vs. *A. bipunctata* confirmed the lower susceptibility of the native species (*A. bipunctata*) to the parasitoid. It has to be stressed that none of the *A. bipunctata* exposed to *D. coccinellae* was successfully parasitized in this experiment, whereas 20% *H. axyridis* produced the parasitoid cocoon. Yet, exposure to parasitoid significantly affected the female reproductive capacity of both species, but especially of *H. axyridis*.

## 5. Conclusions

In conclusion, *D. coccinellae* negatively affected the fitness of *H. axyridis*, more than that of *A. bipunctata*. The parasitoid may thus have a minimal impact on the Asian lady beetle population, without changing the invasiveness status of the exotic coccinellid species. The impact of this parasitoid alone, though marginal, may, however, be higher in biodiversity scenarios where other antagonists, including generalist predators [23] and parasitic fungi [50,51] contribute to limit the population of the coccinellid species. Other factors such as intraguild predation (IGP) can influence coccinellid guild. IGP of *H. axyridis* was demonstrated by field data using molecular gut-content analysis [52]. Many factors can affect the coexistence of exotic vs. native species, including aphid availability [7], the co-presence of other exotic species [53] and the receiving environment [54]. However, a long-term study of coccinellid communities in Central Europe over 35 years, demonstrated that the compositions of the guilds communities remained essentially similar [54].

## Figures and Tables

**Table 1:** Effect of exposure time of *H. axyridis* to *D. coccinellae* on mean (±SE) percentage of parasitoid cocoons detected and of parasitoid adults emerged from cocoons.

Exposure Time	Parasitoid Cocoons Detected ^(1)^	χ^2^ ^(3)^	*p*	Parasitoid Adults Emerged ^(2)^	χ^2^ ^(3)^	*p*
5 min	18 ± 0.04	8.22	0.004	89 ± 0.08	0.21	0.647
1 h	36 ± 0.05			89 ± 0.05		
5 min	18 ± 0.04	3.37	0.067	89 ± 0.08	0.20	0.653
24 h	29 ± 0.05			79 ± 0.08		
1 h	36 ± 0.05	1.12	0.291	89 ± 0.05	0.52	0.473
24 h	29 ± 0.05			79 ± 0.08		

We used the 2 by 2 contingency tables for testing the independence of exposure time (5 min, 1 h and 24 h) and number of parasitoid cocoons detected or parasitoid adults emerged from cocoons. ^(1)^ Percentage calculated on the original number of *H. axyridis* exposed (i.e., 100 per exposure time); ^(2)^ Percentage calculated on the parasitoid cocoons detected (i.e., 18 (5 min), 36 (1 h), 29 (24 h)); ^(3)^ df = 1.

**Table 2:** Development times in days (mean ± SE) of *D. coccinellae* in *H. axyridis*: (I) from exposure to parasitoid cocoon detection; (II) from parasitoid cocoon detection to adult emergence; (III) from exposure to parasitoid adult emergence (total development time).

Exposure Time	Time from Exposure to Cocoon	Time from Cocoon to Adult	Time from Exposure to Adult
5 min	17.44 ± 1.34 a	7.44 ± 0.96	24.68 ± 1.20 a
1 h	17.08 ± 0.77 a	7.25 ± 0.76	24.38 ± 0.87 a
24 h	19.41 ± 3.21 b	7.48 ± 1.2	26.70 ± 3.59 b
	H (N) = 23.66 (83)	F (df) = 0.43 (2,64)	H (N) = 11.34 (71)
*p*	<0.001	0.65	0.035

Means in a column followed by different letters are significantly different (*p* < 0.05; Kruskal-Wallis test followed by non-parametric multiple comparisons).

**Table 3:** Pre-oviposition and oviposition duration in days, total eggs laid in 10 days (E_10_) and fertility (% hatched eggs) (means ± SE) of *H. axyridis* females paired with males not exposed to *D. coccinellae* (controls), exposed and successfully parasitized (successfully exposed) or exposed but not successfully parasitized (unsuccessfully exposed).

Male Parasitization Status	Pre-Oviposition (Days)	Oviposition (Days)	Eggs Laid in 10 Days (E_10_)	Fertility (% Hatched Eggs)
Not exposed (controls) (*n* = 20)	5.25 ± 0.33	20.4 ± 1	306.65 ± 23.32	62.79 ± 3.99 a
Successfully exposed (*n* = 7)	5.86 ± 0.56	19 ± 1.69	310.57 ± 39.31	48.61 ± 6.75 a,b
Unsuccessfully exposed (*n* = 13)	4.85 ± 0.41	20.77 ± 1.24	266.54 ± 28.92	35.06 ± 4.95 b
F (df = 2,37)	1.05	0.37	0.68	9.26
*p*	0.36	0.69	0.51	<0.001

Means in a column followed by different letters are significantly different (*p* < 0.05; one-way ANOVA followed by Tukey’s HSD multiple range test). The number of replicates (*n*) is given in parentheses below the parasitization status.

**Table 4:** Pre-oviposition and oviposition duration in days, total eggs laid in 10 days (E_10_), fertility (% hatched eggs) (means ± SE) of *H. axyridis* females not exposed to *D. coccinellae* (control), exposed and successfully parasitized (successfully exposed), or exposed but not successfully parasitized (unsuccessfully exposed). All females were paired with not exposed males.

Female Parasitization Status	Pre-Oviposition (Days)	Oviposition (Days)	Eggs Laid in 10 Days (E_10_)	Fertility (% Hatched Eggs)
Not exposed (control) (*n* = 20)	7.7 ± 0.47	16.3 ± 0.49 a	359.7 ± 21.22 a	49.92 ± 3.09 a
Successfully exposed (*n* = 3)	8 ± 1.53	8 ± 3.61 b	31 ± 13.08 b	6.06 ± 6.06 b
Unsuccessfully exposed (*n* = 17)	6.59 ± 0.74	11.82 ± 1.51 b	175.53 ± 28.79 b	21.28 ± 4.69 b
	F (2,37) = 0.98	H (40) = 10.76	F (2,37) = 2.19	H (40) = 0.92
*p*	0.38	0.005	<0.001	<0.001

Means in a column followed by different letters are significantly different, *p* < 0.05; one-way ANOVA followed by Tukey’s HSD multiple range test (pre-oviposition and E_10_); Kruskal-Wallis test followed by non-parametric multiple comparisons (other parameters). The number of replicates (*n*) is given in parentheses below the parasitization status.

**Table 5:** Longevity, pre-oviposition and oviposition duration in days, egg fertility (% hatched eggs) of *H. axyridis* and *A. bipunctata* females not exposed (controls) or unsuccessfully exposed to *D. coccinellae* as related to the combination of the factors “coccinellid species” and “exposure to *D. coccinellae*” (means ± SE). All females were paired with non- exposed males.

Parameters	Coccinellid Species	Exposure to *D. coccinellae*		Source of Variation		
		Yes	No	Species Effect	Exposure Effect	Interaction Species × Exposure
Longevity (days)	*H. axyridis*	(16) 20.1 ± 0.9	(20) 22.2 ± 0.9			
				F = 0.02	F = 2.97	F = 0.23
				df = 1,72	df = 1,72	df = 1,72
				*p* = 0.9	*p* = 0.09	*p* = 0.63
	*A. bipunctata*	(20) 20.7 ± 1.2	(20) 21.9 ± 0.8			
Pre-oviposition (days)	*H. axyridis*	(14) 5.93 ± 0.97	(19) 5.11 ± 0.4			
				F = 8.99	F = 1.3	F = 0.34
				df = 1,67	df = 1,67	df = 1,67
				*p* = 0.004	*p* = 0.26	*p* = 0.56
	*A. bipunctata*	(19) 4.21 ± 0.3	(19) 3.95 ± 0.3			
Oviposition (days)	*H. axyridis*	(14) 10.1 ± 1.7	(19) 16.6 ± 0.72			
				F = 3.36	F = 14.4	F = 0.36
				df = 1,67	df = 1,67	df = 1,67
				*p* = 0.07	*p* = 0.0003	*p* = 0.56
	*A. bipunctata*	(19) 12.9 ± 1.5	(19) 17.1 ± 0.9			
Fertility (% hatched eggs)	*H. axyridis*	(14) 22 ± 5.4	(19) 35.4 ± 4.1			
				F = 7.36	F = 6.75	F = 0.39
				df = 1,67	df = 1,67	df = 1,67
				*p* = 0.008	*p* = 0.012	*p* = 0.54
	*A. bipunctata*	(19) 13.7 ± 3.3	(19) 20.6 ± 3.7			

The number of replicates (*n*) is given in parentheses above the means.
